# Modulation of Inflammation-Induced Tolerance in Cancer

**DOI:** 10.3389/fimmu.2020.01180

**Published:** 2020-06-26

**Authors:** Vladimir Rogovskii

**Affiliations:** Department of Molecular Pharmacology and Radiobiology, Pirogov Russian National Research Medical University, Moscow, Russia

**Keywords:** cancer, inflammation, immune tolerance, immune suppression, anti-inflammatory therapy

## Introduction

Chronic inflammation is widely accepted to be an important cause of tumor formation and development (1, 2).

The primary significance of chronic inflammation in cancer may be in its linkage to immune tolerance. According to Peter Medawar, “‘Immunological tolerance’ may be described as a state of indifference or non-reactivity toward a substance that would normally be expected to excite an immunological response” (3).

During acute inflammation, immune suppression is a temporary stage toward the resolution of inflammation (4). However, in the case of chronic inflammation, this stage becomes permanent, leading to true immune suppression and immune tolerance. Thus, chronic low-grade inflammation may be the universal mechanism of immune tolerance—both in disease and in health (2). According to this concept, immune tolerance may be the physiological counterpart to chronic low-grade inflammation [so-called para-inflammation (5)]. This is the case for organs with elevated immune tolerance, e.g., gut, placenta, brain, testis—they have a normally elevated level of several inflammatory factors (2, 5–8).

There are various reasons for chronic inflammation: e.g., prolonged contact with irritating factors (physical or chemical), chronic infections, chronic stress, insufficient physical activity, obesity, disturbances in gut microbiota, and an “inflammatory diet” (e.g., a western diet high in processed meat and fat but with low fiber and low omega 3/omega 6 fatty acid ratio) (2, 5, 9, 10). Moreover, in the case of an advanced tumor, such tumors promote an inflammatory environment *per se*, leading to chronic inflammation (2).

**Figure 1:**
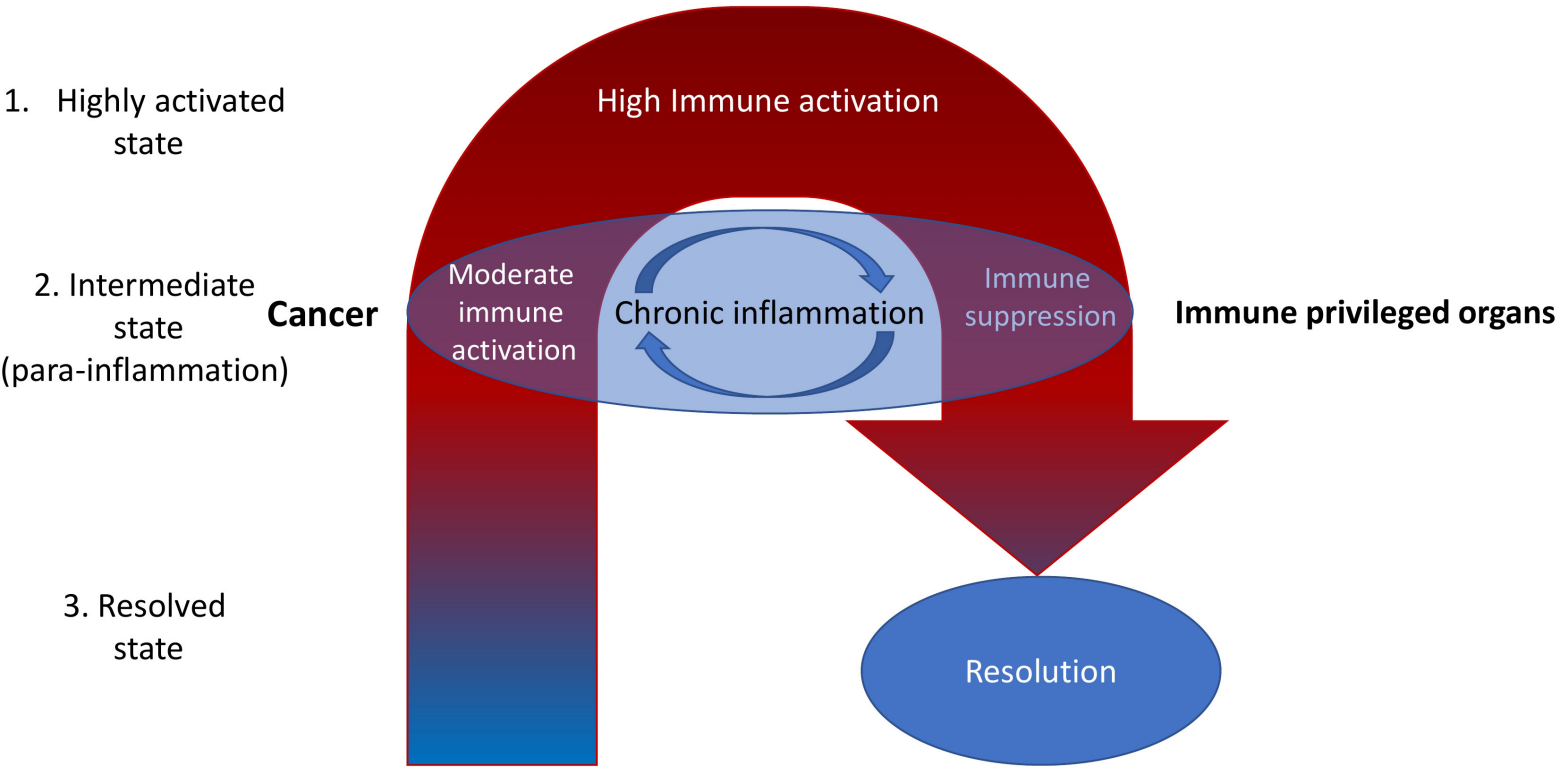
It is reasonable to highlight the three types of inflammatory microenvironments: highly activated state, intermediate state, and the resolved state. Cancer is often associated with the intermediate state [para-inflammation (6)]. Most current therapeutic efforts are directed toward increasing inflammation toward the highly activated state. However, this is often associated with internal compensative mechanisms resulting in subsequent immune suppression (negative feedback). That may be the reason for the limited efficiency of ICIs, which dramatically increase inflammation at the whole-organism level (11). A better way may be to reduce inflammation with the aim of subsequent activation of the normal process of immune rejection of tumors (fast and without entering the cycle of chronic inflammation)—it might be called the immunological reload. Nevertheless, there still may be non-immune factors in the tumor microenvironment that decrease the immune response. For example, high levels of oxidized low-density lipoproteins and lactate (12). They also should be eliminated.

There might be two ways of mitigating chronic inflammation in cancer—intensification of inflammation and suppression of the inflammation. In the case of increasing the inflammation, we intend to surpass inflammation-related tolerance. In the case of suppressing the inflammation, we intend to suppress inflammation-related tolerance, after which there might be tumor rejection (Figure 1).

## Cancer-Related Inflammation and Immune Tolerance

Tumors produce several inflammation-related factors, for example, IL-6, IL-8, IL-10, CCL2, and CCL5 (1, 13, 14).

The current paradigm of the antigen-specific immune suppression is the action of myeloid-derived suppressor cells (MDSCs) (15). According to much studies, this heterogenic cell population may include various dendritic cells, macrophages (M2 macrophages), monocytes and plasmacytoid cells having a similar main feature. They can present antigens to lymphocytes inducing the antigen-specific immune suppression in health (e.g., in immune privileged organs) and disease (16–18). These heterogeneous cell groups express various markers, but the markers from the number of CD11b, CD11c, and CD33 might be the most typical of them (14, 18). Lechner et al. revealed that induction of CD33+ MDSCs and CD11b+ MDSCs depended upon over-expression of various cytokines by a range of different tumor cell lines [especially IL-1β, IL-6, TNFα, VEGF, GM-CSF for CD33+ MDSCs and FMS-like tyrosine kinase 3 ligand (FLT3L), and TGFβ for CD11b+ MDSCs] (14).

In cancer, MDSCs shape and promote metastatic dissemination (15). Moreover, a low level of MDSCs in patients with cancer has been shown to be correlated with their good prognosis after immune checkpoint inhibitors treatment (19). Among the mechanisms of MDSCs-mediated immune suppression are the production of reactive oxygen species, NO, arginase (Arg-1), indoleamine oxidase (IDO), and immunosuppressive cytokines, such as IL-10, TGF-β, and T-regulatory cells (Tregs) expansion (20, 21).

## Increasing Cancer-Related Inflammation

The first methods for treating cancer by increasing inflammation were made over a century ago by William Coley and many of his followers. In 1891, William Coley injected streptococcal organisms into a patient with inoperable cancer. It was successful, and this was one of the first examples of cancer immunotherapy that enhanced the immune system (22). Another step in the research of increasing cancer-related inflammation was made about 80 years ago by Roskin and colleagues by using *Trypanosoma cruzi* (the protozoan agent of Chagas' disease). It was proposed that this infection mediates an adaptive immune response with significant antitumor effects (23).

Many attempts were made later to promote the antitumor immune response by increasing inflammation using other approaches.

In particular, cytokine therapy [e.g., IL-2, interferon (IFN)-α], antitumor vaccines, and even oncolytic viruses (e.g., genetically modified type I herpes simplex virus) were used. Currently, the most widespread way of increasing cancer-related inflammation is the use of immune checkpoint inhibitors (ICIs) and chimeric antigen receptor T (CAR-T) cells (11, 12, 24–28).

One of the most commonly used cytokines in cancer treatment is IL-2. IL-2 promotes antigen-activated CD8+ T-cells and is a growth factor for CD4+ T-cells as well as NK-cells. IL-2 has some proven efficacy in treating metastatic renal cell carcinoma and metastatic melanoma (MM) patients (26). The meta-analysis showed that IL-2 immunotherapy for advanced MM delivered a complete response rate of 4% (29). Besides the immune stimulation, IL-2 plays an essential role in immune suppression as part of a negative feedback loop cascade. IL-2 is a potent negative immune regulator stimulating immunosuppressive Tregs (26). That immune-suppressive role of cytokine signaling might be the cause of poor clinical results.

Oncolytic viruses are another type of inflammation-enhancing immune therapy. They are designed to target and kill cancer cells, leaving normal cells unaffected (30). For example, the modified oncolytic Herpes simplex virus 1 (HSV-1), talimogene laherparepvec (T-VEC), has been shown to suppress the growth of advanced malignant melanoma in humans (31). It is the first approved oncolytic virus in the USA (2015). In the phase III trial of T-VEC, the objective response rate and complete response rate were 26 and 11%, respectively, compared to 6 and 1% for recombinant GM-CSF (32). Besides the direct killing of cancer cells, oncolytic viruses can modulate the tumor microenvironment toward a more inflammatory phenotype and induce anti-cancer immunity (30). These processes are very complicated, as there are multiple negative feedback mechanisms. For example, it was shown that chronic viral infection could enhance NK-cells function. This effect is mediated by type I IFN signaling, and it can lead to the killing of virus-specific T cells. The biological sense of this is to minimize T-cell-mediated pathologic damage (33). Minimizing the T-cell-mediated response can limit cancer cell killing by T cells.

It should be taken into account that any induction of inflammatory phenotype leads to a compensatory anti-inflammatory and immune-suppressive response sooner or later. In that stage, after the initial reduction of tumor volume, cancer cells might start to proliferate more extensively.

ICIs block signaling through inhibiting receptors in immune cells. The first checkpoint inhibitor was approved in 2011, opening a new era in cancer immunotherapy. Typically, ICIs increase inflammation at the whole organism level (34). This increment at the initial stage can be associated with increased inflammation-related immune tolerance and might be the reason for tumor pseudoprogression. After the predomination of the immune-inflammatory process over immune tolerance, there may be clinical remission.

It should be noted that there are multiple mechanisms of negative feedback in immunity, such as MDSCs, Tregs, and many immune checkpoints (besides CTLA-4 and PD-1, there are TIM-3: mucin-domain-containing protein-3, LAG-3: lymphocyte-activated gene-3, and many others). Moreover, this potent immune-suppressive machinery tends to be activated by increased ICIs-mediated or CAR-T-mediated immune inflammation. That might be the reason why, after an initial response to checkpoint blockade, acquired resistance occurs in most patients (35). The phenomenon of hyperprogression (paradoxical acceleration in tumor growth observed in certain patients following the administration of immune checkpoint inhibitors) also can be linked to these mechanisms (34). In line with them, it was recently found that the percentage of CD8-T-cells that express LAG-3 and PD-1 was significantly increased in the dysfunctional response group to CAR T-cell therapy (36).

## Reducing Cancer-Related Inflammation

Mechanisms of resolution of inflammation are of vital importance for cancer prevention. Animals lacking in immunosuppressive mediators show chronic inflammation and increased cancer frequency (37, 38). Anti-inflammatory strategies for cancer treatment include the use of all-trans-retinoic acid (ATRA), vitamin D, non-steroidal anti-inflammatory drugs (NSAIDs), several anti-inflammatory antibodies, etc.

ATRA is the primary biologically active metabolite of vitamin A that possesses anti-inflammatory properties (39). ATRA is crucial for dendritic cells to facilitate the generation of Tregs and suppress the differentiation of naive CD4+ cells into inflammatory Th17-cells (40). ATRA also influences the maturation of MDSCs by increasing the expression of major histocompatibility complex class II and CD86 (41).

It is reasonable to suppose that termination of inflammation (resolution) should also cause the termination of the action of immune-suppressive mechanisms.

For instance, it was shown that *in vitro* treatment with ATRA decreases the immunosuppressive function of MDSCs in mixed lymphocyte reactions. ATRA also reduces the expression of immunosuppressive genes, including PD-L1, IL-10, and IDO, by MDSCs. In a randomized phase II clinical trial, ATRA significantly decreased the frequency of circulating MDSCs compared to ipilimumab treatment alone in advanced-stage melanoma patients (42). Currently, there are several ongoing trials exploring ATRA in cancer treatment (43, 44).

Vitamin D (e.g., calcitriol) exhibits the anti-inflammatory actions that may contribute to its beneficial effects in several cancers. Calcitriol is involved in the inhibition of the synthesis of prostaglandins, suppression of stress-activated kinase signaling, suppression of NF-κB signaling, and other anti-inflammatory mechanisms (45). MDSCs are considered to be one of the important vitamin D targets. It was shown that vitamin D treatment reduced the T-cell suppressive capacity of cytokine-induced MDSCs by ≥70% (46).

There are various data concerning vitamin D and cancer incidence and treatment. For instance, it was shown that vitamin D was associated with a significant reduction of cancer-related mortality compared with placebo (response rate 0.87) (47).

Polyphenols and related compounds are plant metabolites that contain multiple phenolic groups. Polyphenols possess various anti-inflammatory properties. Due to their molecular structure, they can serve as hydrogen bond donors to many proteins and nucleic acids. That is why there are various molecular targets of polyphenols, mainly in the signal transduction pathways (48, 49).

For instance, in prostate cancer cell lines, polyphenol resveratrol reduced the levels of several receptor tyrosine kinases (e.g., epidermal growth factor receptor) (50). Catechins (the water-soluble polyphenolic substances found in green tea) also can interact with the ATP binding site of some receptor tyrosine kinases and inhibit tyrosine phosphorylation (48, 51). Resveratrol induces autophagy by directly inhibiting mTOR through ATP competition (52).

According to a recent 5-year trial, oral administration of indole-3-carbinol [plant metabolite, obtained from cruciferous vegetables (53)] and epigallocatechin-3-gallate (probably the most active of catechins) as maintenance therapeutic agents in advanced ovarian cancer has demonstrated a dramatic increase in median progression-free survival (approximately double) (54).

Of course, we should discuss the most widely used anti-inflammatory drugs—NSAIDs (e.g., aspirin). Their ability to prevent cancer has been known for years. According to a 2018 meta-analysis, a protective effect was identified for the intake of any NSAIDs against the risk of prostate cancer (55). According to another recent study, regular use of cyclooxygenase-2 inhibitors was associated with a 71% reduced risk of breast cancer (56). NSAIDs are involved in modulation of the synthesis of various eicosanoids. It is not just inhibition, but a more complicated process during which synthesis increases of cyclooxygenase- and lipoxygenase-derived anti-inflammatory factors like lipoxin A4, resolvin D1, etc. Eicosanoid mediators are involved in the induction and maintenance of immune tolerance. For instance, lipoxin A4 is involved in tolerance induction during allergen immunotherapy (57). According to a recent study, colorectal cancer is associated with a deficiency of lipoxin A4 (58). In murine xenograft tumor models, lipoxin A4 is able to suppress tumor growth by targeting immune-suppressive IL-10-producing regulatory B cells via dephosphorylation of STAT-3 and extracellular signal-regulated kinase (59).

## Conclusion and Prospects

The efficacy of inflammation-increasing immunotherapy depends on the cancer type (more suitable for “hot” tumors, i.e., those that show signs of inflammation) and is limited by major side effects. Active inflammation is often associated with internal compensative mechanisms resulting in possible subsequent immune suppression—it may be one of the reasons for the low response to tumor immunotherapy.

Much data exists regarding the efficiency of suppressing cancer-related inflammation. The aim of this suppression should be to abrogate immune tolerance associated with chronic inflammation. It is probably reasonable to study combination therapy: anti-inflammatory therapy with immunotherapy such as ICIs and CAR-T cells. Such a combination seems contradictory. However, just as one often needs to combine different directions to reach a certain point, e.g., turn left and turn right depending on the current route, cancer immune therapy could require a similar twist.

## Author Contributions

VR conceived and wrote the article, read, and approved the submitted version.

## Conflict of Interest

The author declares that the research was conducted in the absence of any commercial or financial relationships that could be construed as a potential conflict of interest.
